# The Bimanual Signs of Early Pregnancy

**Published:** 1892-11

**Authors:** 


### THE BIMANUAL SIGNS OF EARLY PREGNANCY.

Dr. R. L. Dickinson (*New York Journal of Gynecology and Obstetrics*) says:

The presence or absence of pregnancy may be determined in favorable cases by bimanual examination between the second and sixth week after coitus, or between the third and eighth after the beginning of the last menstruation.

Bellying or bulging of the surface of the body of the uterus is the most constant and valuable sign. It is rarely absent (4 per cent.) It may usually be found by the twenty-eighth day after coitus, although often present by the sixteenth or twenty-second day. Occurring most frequently on the anterior face (40 per cent.), it may appear on both, while in retroversion it is found posteriorly, and in certain cases laterally.

Elasticity or resiliency of the body of the uterus is more readily detected than the above, but less frequently present (80 per cent.). It is present, on an average, by the thirtieth day after coitus, but occasionally found by the sixteenth or twenty-first day. Usually it appears later than bellying.

Compressibility of the lower uterine segment (Hegar's sign) is less often found than the two preceding (66 per cent.); when it appears it may be fairly well defined by the twenty-fourth day after intercourse, but is often indistinct until the thirtieth or fiftieth. It is, therefore, a later sign than the preceding.

A traverse fold on the anterior uterine wall is usually distinct in the relaxed condition. Although infrequently found, this sign is of great value.—*The American Lancet*; *Cin-Lancet-Clinic.*

